# A cross-sectional study among Polish hunters: seroprevalence of hepatitis E and the analysis of factors contributing to HEV infections

**DOI:** 10.1007/s00430-017-0515-0

**Published:** 2017-08-03

**Authors:** Anna Baumann-Popczyk, Bartłomiej Popczyk, Elżbieta Gołąb, Wioletta Rożej-Bielicka, Małgorzata Sadkowska-Todys

**Affiliations:** 1 Department of Epidemiology, National Institute of Public Health-National Institute of Hygiene, Chocimska str 24, 00-791 Warsaw, Poland; 2 Department of Genetic and Animal Breeding, Faculty of Animal Science, University of Life Sciences, Warsaw, Poland; 3 Polish Hunting Association, Warsaw, Poland; 4 Department of Parasitology, National Institute of Public Health-National Institute of Hygiene, Warsaw, Poland

**Keywords:** Epidemiology, Foodborne viruses, Zoonosis, Hunters, Hepatitis E (HEV)

## Abstract

Hepatitis E virus (HEV) is known as zoonotic agent. The main reservoirs of HEV in Europe are pigs, wild boars, and deer. Hunting activity is considered to be a risk factor for HEV infection. We conducted a cross-sectional study among 1021 Polish hunters. To understand socio-demographic characteristics of this population and to gather information on potential exposures, all participants completed a questionnaire. Commercial immunoassays were employed to estimate seroprevalence anti-HEV. Samples with confirmed positive result of anti-HEV IgM were examined for HEV RNA. Anti-HEV IgG were identified in 227 people, 22.2% of the studied group. Seroprevalence among the studied hunters was associated with age ≥65 [adjusted prevalence ratio (aPR) 1.6, *p* = 0.037), living in a house (aPR 1.54, *p* = 0.013), professional contact with farm animals (aPR 1.09, *p* = 0.01), and consumption of stewed offal (aPR 1.61, *p* = 0.00). Washing hands after disembowelment was linked to lower seroprevalence (aPR 0.53; *p* = 0.00). Lower prevalence of anti-HEV IgG among hunters living in cities was associated with age: 35–49 (aPR 0.52, *p* = 0.011) and 50–64 (aPR 0.93, *p* = 0.58), living in a house (aPR 1.58, *p* = 0.002) and owning a cat (aPR 0.58, *p* = 0.042). Among hunters living in rural areas, seropositivity was associated with contact with farm animals (aPR 1.66, *p* = 0.013) and consumption of stewed offal (aPR 1.81; *p* = 0.001). Contrary to initial assumptions, it was concluded that hunting was of significantly lesser importance than other factors. Due to the high level of HEV seroprevalence identified, we recommend conducting a large-scale study in the general population of Poland.

## Introduction

The hepatitis E virus (HEV) is an important human pathogen that is distributed worldwide [1]. In the last few years, HEV has been considered as an emerging infection or, by some researches, as a re-emerging disease [2]. Hepatitis E virus (HEV) is a non-enveloped RNA virus in the genus *Orthohepevirus* in the family Hepeviridae. Genotypes 1 (HEV-1) and 2 (HEV-2) cause infections in developing countries. Those infections are restricted to humans and transmitted by the oral-fecal route mainly through contaminated water [1]. Genotypes 3 (HEV-3) and 4 (HEV-4) have a wider host range, including humans and various animal species [3]. It was proved that those genotypes cause sporadic cases of hepatitis E in humans, mostly through zoonotic transmission or consumption of contaminated food [3, 4]. Alternative modes of transmission of minor epidemiological importance, such as blood transfusion [5] and organ transplantation [6, 7], were also reported. HEV-3 genotypes were found to be responsible for most autochthonous HEV infections in Europe, and to the lesser extent, infections with HEV-4 were also noted [8, 9].

Diseases caused by HEV genotypes present clinical features similar to those occurring during infections caused by other hepatitis viruses. The course of infection can involve a wide range of clinical manifestations, from asymptomatic or subclinical to acute liver failure [10]. Chronic infections with HEV-3 and HEV-4 were found in immunocompromised patients, such as solid organ transplant recipients and HIV carriers [11]. Extra-hepatic manifestations, such as haematological and neurological disorders, kidney injuries, or acute pancreatitis, can also be associated with the HEV infection [12].

Seroprevalence of HEV in Europe in human ranges from 0.6 to 52.5%, depending on the country, study group, and type of diagnostic test [8, 13]. Domestic pig, wild boar, and deer were identified as major reservoirs of zoonotic HEV in Europe in many studies [3]. The highest HEV seroprevalence, ranging from 30 to 98%, was observed in swine herds [14], whilst among tested wild boars, it reached the maximum of 47.2% [15]. HEV-3 was isolated from infected swine and wild boars [3]. Occupational exposure to swine [applying to veterinarians, slaughterhouse workers, and farmers) is associated with higher prevalence of anti-HEV IgG, what supports the zoonotic nature of HEV infections [16].

Although HEV infections are a cause for concern, the epidemiological situation is not under comprehensive control in Poland. This may be due to the lack of routine HEV diagnostics and low awareness among doctors of this virus and infection, which often takes nonspecific or asymptomatic course. In Poland, hepatitis E is a notifiable disease listed among “infections caused by other hepatitis viruses”—there is no case definition of hepatitis E. In the years 2014–2015, eight human cases of hepatitis E were notified and most of them were laboratory confirmed outside of Poland (source: Department of Epidemiology at NIPH–PZH). There was found only one case report on hepatitis E diagnosed in Poland in foreign student residing in north-eastern part of our country [17].

The precise data on seroprevalence of HEV in the general population in Poland are limited. However, several results of HEV seroprevalence surveys have been published. One of them was conducted among Indian citizens who studied in Polish university located in eastern part Poland and serological markers of HEV infection were detected in 12 (26.7%) tested students [18]. In the study conducted in the west-central Poland IgG, anti-HEV was found in 15.9% within 182 tested serum samples [19]. In the above mentioned region, 50.2% HEV-positive results were obtained in a study group consisted of HIV-infected persons and blood donors [20].

There are evidences that HEV circulates among domestic pigs, wild boar, and this species should be considered as important reservoirs of the virus in Poland [21–23]. Population of wild boar in Poland is growing, from 120,000 in 1999/2000–264,000 in 2015/2016, and currently, it is one of the most intensively hunted species in our country (Central Statistical Office, 2016). As a result of frequent and direct contact with infected wild animals, hunters are potentially exposed to zoonotic pathogen [24]. According to data from the Polish Hunting Association, 10% of its members work in agriculture, including pig farming, which is a known risk factor for HEV infection [16].

In Europe, there have been reports on the increasing prevalence of HEV among people as well as an increasing number of reports on negative health consequences of being infected [9, 11]. Therefore, the need to investigate the epidemiological profile of HEV in Poland is a necessity.

The purpose of this study was to assess HEV seroprevalence in the population of Polish hunters. Furthermore, the study aimed to provide in-depth analysis of the data gathered in previous study to identify factors associated with the prevalence of anti-HEV IgG antibodies in the examined population.

## Materials and methods

### Study design

The presented cross-sectional study was carried out between October 2010 and July 2012 among Polish hunters. The study was a part of a larger project financed by the Ministry of Science and Higher Education (N N404 520038) entitled “Occurrence and prevalence of selected zoonotic agents: *Echinococcus multilocularis*, *Trichinella spiralis* and hepatitis E virus (HEV) in the population of Polish Hunters” [25].

For the purpose of this study, a hunter was defined as a member of the Polish Hunting Association (PHA), in accordance with Polish law (the Hunting Act of October 13, 1995). The study was approved by Bioethics Commission in the National Institute of Public Health–National Institute of Hygiene in Warsaw (Opinion no 1/2009 of 15.10.2009).

Authors of the study invited hunters from all regional units of the Polish Hunting Association. The study participants’ recruitment was conducted through convenience sampling, in line with guidelines previously described by Sadkowska-Todys et al. [25]. Participation in the study was voluntary. Potential participants were provided with information on the aim of the study, as well as a leaflet about the study and pathogens that they might be tested for. Written consent was obtained from all recruited participants. Socio-demographic characteristics (e.g., age, gender, and place of residence) and information on potential exposures (e.g., area of hunting, length of membership of the PHA, preparation and consumption of wild game meat, etc.) were collected using structured questionnaires administered through face-to-face interviews. The study questionnaire was designed in the Department of Epidemiology after consultation with the PHA and piloted among 91 hunters. Information on history of exposures was assessed through simple closed questions. Previous exposures related to hunting were ranked based on hunting activities or preferences.

### Data on general hunter population in Poland

The Central Statistical Office of Poland (Forestry 2012) and the PHA provided data on the population and characteristic of Polish hunters.

### Laboratory testing

To identify serological markers of infection, in our study, we used two commercial immunoenzymatic tests: recomWell HEV IgG and recomWell HEV IgM (versions available in Poland in 2011; Mikrogen, Neuried, Germany). Each anti-HEV IgM antibody positive EIA test result was confirmed with the recomLine HEV IgM/IgG immunoblot test (version available in Poland in 2011; Mikrogen, Neuried, Germany).

All positive samples in the immunoblot test were examined for the presence of HEV RNA. The real-time PCR reaction was performed using the commercial ampliCube HEV 2.0 set (Mikrogen, Neuried, Germany).

Tests were carried out in accordance with the manufacturer’s instructions.

### Statistical analysis

Prevalence of anti-HEV antibodies (IgG and IgM) was calculated and the exact binomial distribution was used to calculate 95% confidence intervals (CI).

Possible predictors of anti-HEV seropositivity were assessed by calculating prevalence ratio (PR) and adjusted prevalence ratio (aPR) and their corresponding 95% confidence intervals (CI), using univariable and multivariable log binomial regressions, respectively.

In this study, a case was defined by the positive result of recomWell IgG. The final multivariable model was built stepwise using results of univariate analysis (where *p* ≤ 0.2). In addition, to differentiate specific predictors for infection between urban and rural areas, the studied group was stratified by place of residence. Data were analyzed by the statistical package Stata 10.

## Results

### Characteristics of the study group

A total number of 1021 from 1041 recruited participants met our case definition of a hunter. The study sample represented 0.9% of the hunters’ population in Poland. Participants were recruited through convenience sampling. Therefore, the first stage of the study compared selected features of the examined group with the entire hunters’ population. The general characteristics, such as gender, age or length of membership in the PHA, were the same or very similar to those in the general hunter population in Poland (Table 1).

**Table 1:** Comparison of participants to the source population of Polish hunters by gender, age category, and place of residence

Characteristics	Source population^a^		Participants	
	*N*	%	*N*	%
Total	111,948	100	1021	0.9
Sex				
Female	2614	2.3	23	2.3
Male	109,334	97.7	998	97.7
Age category (years)				
>30	9101	8.1	91	8.9
31–40	19,602	17.5	182	17.8
41–50	21,069	18.8	217	21.3
51–60	26,420	23.6	312	30.6
61–70	23,845	21.3	181	17.7
<71	11,766	10.5	38	3.7
nd	146	0.1	–	–
Length of membership of the PHA (in years)				
0–3	15,079	13.5	65	6.4
4–10	23,050	20.6	191	18.7
11–20	23,957	21.4	247	24.2
21–30	23,509	21	278	27.2
>31	26,140	23.4	240	23.5
nd	213	0.2	–	–
Province				
Dolnośląskie	8932	8	60	5.9
Kujawsko-Pomorskie	6537	5.8	50	4.9
Lubelskie	6870	6.1	149	14.6
Lubuskie	4925	4.4	6	0.6
Łódzkie	5520	4.9	27	2.6
Małopolskie	6896	6.2	48	4.7
Mazowieckie	13,902	12.4	117	11.5
Opolskie	3550	3.2	59	5.8
Podkarpackie	6756	6	34	3.3
Podlaskie	4515	4	44	4.3
Pomorskie	6953	6.2	44	4.3
Śląskie	6512	5.8	38	3.7
Świętokrzyskie	3663	3.3	36	3.5
Warmińsko- Mazurskie	6886	6.2	45	4.4
Wielkopolskie	11,575	10.3	105	10.3
Zachodniopomorskie	7956	7.1	159	15.6

^a^ Members of the Polish Hunting Association in 2011

Participants’ age ranged from 19 to 85 years (mean 49.5 years). The most frequent group of respondents were persons aged 51–60. The average age was higher among men (mean 49.8, median age 51 years) than among women (mean 37.2, median 30 years). However, those differences were not statistically significant.

### Prevalence of HEV in study group

Anti-HEV IgG were found in 227 people, which represented 22.2% of the studied group (95% CI 19.7–24.9).

Anti-HEV IgM were detected in 43 individuals in recomWell IgM test (4.21% of the examined group of hunters). Using recomLine IgM/IgG, anti-HEV IgM were found in five of the hunters. This represented 0.5% of the entire studied group. HEV RNA was not detected in the serum of persons with confirmed anti-HEV IgM. None of those persons had symptoms of acute hepatitis.

### Factors associated with anti-HEV IgG seroprevalence

**Fig. 1:**
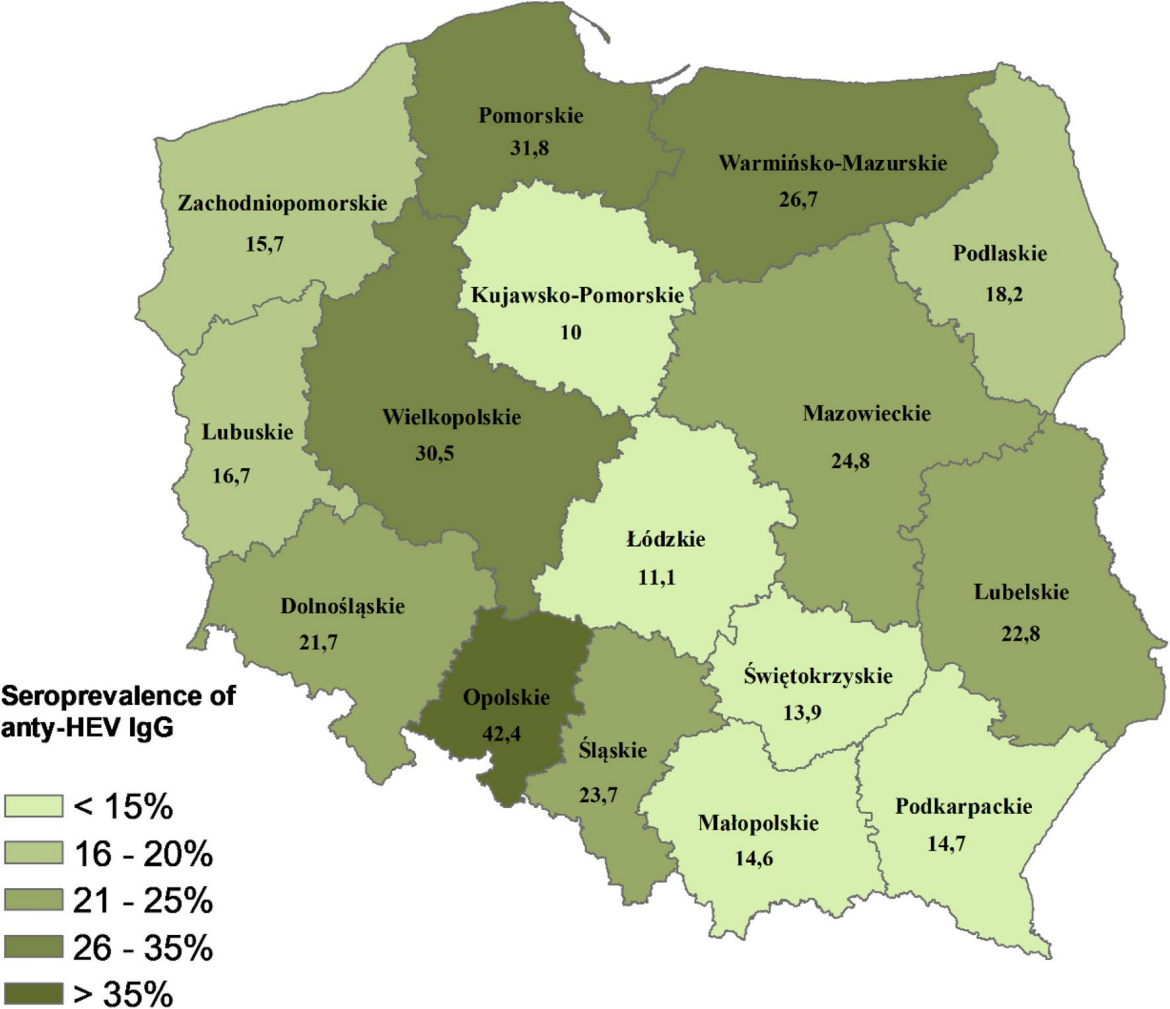
Seroprevalence of anti-HEV IgG among hunters by the province of residence

Significant differences in the number of anti-HEV IgG positive cases in the studied group were observed depending on the province. The highest seroprevalence was among hunters who lived in Opolskie (42.4%), Wielkopolskie (30.5%), and Pomorskie (31.9%). The lowest was recorded among people living in Kujawsko-Pomorskie (10%), Łódzkie (11.2%), and Świętokrzyskie (13.9%) (Fig. 1).

Multivariable regression analysis confirmed that the prevalence of HEV infection increased with age. People aged over 65 years had anti-HEV IgG significantly more frequently than those who were younger [adjusted prevalence ratio (aPR) 1.6; 95% CI 1.03–2.49]. The percentage of HEV positivity was 40% higher among hunters living in a house than those living in an apartment (aPR 1.4; 95% CI 1.09–2.01). Those who declared direct contact with farm animals, mainly pigs, had anti-HEV antibodies 40% more often (aPR 1.41; 95% CI 1.09–1.83). Moreover, washing hands immediately after disembowelment reduced the risk of infection (aPR 0.53; 95% CI 0.37–0.74). A higher seroprevalence was also found among hunters who hunted wild boars three times a year (on a five-point scale) compared to those who hunted wild boar once a year. Consuming stewed offal, e.g., liver, was linked to higher seroprevalence in the studied group (aPR 1.61; 95% CI 1.27–2.03) (Table 2).

**Table 2:** Seroprevalence of anti-HEV IgG and risk factors associated with HEV infection among Polish hunters

Variable	No. of subjects	No. of positive (%)	95% CI	PR (95% CI)	*p*	APR (95% CI)	*p*
Age (years)							
≤34	174	36 (20.69)	14.93–27.47	Reference		Reference	
35–49	294	58 (19.73)	15.33–47.43	0.95 (0.66–1.38)	0.802	0.8 (0.63–1.01)	0.060
50–64	442	99 (22.40)	18.59–26.58	1.08 (0.77–1.52)	0.646	0.91 (0.67–1.22)	0.494
≥65	111	34 (30.63)	22.23–40.09	1.48 (0.99–2.21)	0.057	1.6 (1.03–2.49)	**0.037**
Type of accommodation							
Flat	315	51 (16.2)	12.30–20.73	Reference		Reference	
House	706	176 (24.9)	21.78–28.29	1.54 (1.61–2.04)	**0.003**	1.48 (1.09–2.01)	**0.013**
Occupation							
Employment in the private sector	84	15 (17.9)	10.35–27.74	Reference		Reference	
Unemployed	6	3 (50)	11.81–88.19	2.8 (1.11–7.04)	0.029	1.86 (0.53–6.58)	0.339
Retiree and pension	193	49 (25.4)	19.41–32.14	1.42 (0.85–2.39)	0.183	1.08 (0.6–1.95)	0.799
Other	280	53 (18.9)	14.51–24.02	1.06 (0.63–.78)	0.826	1.03 (0.6–1.76)	0.926
Woodsman	167	37 (22.2)	16.10–29.22	1.24 (0.72–2.13)	0.433	0.98 (0.56–1.72)	0.941
Scientific worker	26	7 (26.9)	11.57–47.79	1.51 (0.69–3.29)	0.303	1.46 (0.66–3.27)	0.361
Working in PHA	55	9 (16.4)	7.76–28.80	0.92 (0.43–1.95)	0.820	0.86 (0.38–1.93)	0.702
Farmer	57	19 (33.3)	21.40–47.06	1.87 (1.04–3.36)	0.037	1.22 (0.66–2.27)	0.531
Student	12	2 (16.7)	2.09–48.41	0.93 (0.24–3.58)	0.920	0.72 (0.19–2.73)	0.627
Company owner	114	28 (24.6)	16.98–47.16	1.38 (0.79–2.41)	0.265	1.06 (0.58–1.95)	0.853
Army and police	21	5 (23.8)	8.22–47.16	1.33 (0.55–3.25)	0.527	0.88 (0.28–2.79)	0.816
Professional contact with animals (*n* = 952)							
No	796	154 (19.3)	16.89–22.53	Reference		Reference	
Yes	156	71 (45.5)	25.54–38.06	1.61 (1.27–2.04)	0	1.09–1.83	**0.01**
Hunting abroad in the last 14 years							
No	890	200 (22.5)	19.77–25.36	Reference		Reference	
Yes	129	22 (17.1)	11.01–24.67	0.76 (0.51–1.13)	0.176	0.45–1.12	0.134
Hand washing after disembowelling (*n* = 901)							
No	30	10 (33.3)	17.29–52.81	Reference		Reference	
Yes	871	186 (21.4)	18.68–24.23	0.64 (0.38–1.09)	0.094	0.37–0.74	**0.000**
Cultivating the cereals (*n* = 1014)							
No	738	154 (20.9)	18.00–23.98	Reference		Reference	
Yes	276	72 (26.1)	21.00–31.69	1.25 (0.98–1.60)	0.072	0.86–1.39	0.507
Presence of rodents in the place of accomodation (*n* = 1012)							
No	363	67 (18.5)	14.35–22.54	Reference		Reference	
Yes	649	159 (24.5)	10.94–27.68	1.33 (1.03–1.71)	0.029	0.84–1.48	0.468
Average annual wild boars hunting (on a five-point scale) (*n* = 984)							
1	101	17 (16.8)	10.12–25.58	Reference		Reference	
2	130	37 (28.5)	20.90–37.04	1.69 (1.01–2.82)	0.044	1.36 (0.79–2.35)	0.271
3	171	37 (21.6)	15.71–28.57	1.29 (0.77–2.16)	0.343	1.66 (1–2.75)	**0.054**
4	212	54 (25.5)	19.75–31.89	1.51 (0.93–2.47)	0.098	1.45 (0.88–2.4)	0.152
5	370	79 (21.4)	17.28–25.88	1.27 (0.79–2.04)	0.327	1.17 (0.72–1.9)	0.551
Prepared and consumed stewed offal (*n* = 985)							
No	839	176 (21)	18.27–23.89	Reference		Reference	
Yes	146	45 (30.8)	23.45–38.99	1.47 (1.11–1.94)	0.006	1.61 1.27–2.03	**0.000**

Significant* P* values (< 0.05) are in bold

PR (prevalence ratio), APR (adjusted APR for age, place of accommodation, occupation, professional contact with animals, hunting abroad, hand washing after evisceration, cultivating the cereals, presence of rodents in the place of accommodation, prepared, and consumed stewed offal)

Based on our preliminary results, we stratified the examined hunters by place of residence to identify specific risk factors for urban and rural areas.

Prevalence of anti-HEV IgG among hunters living in cities was also linked with age. The frequency of HEV IgG antibodies was significantly lower among hunters aged 35–49 and 50–64 than hunters aged up to 34 years (aPR 0.52; 95% CI 0,31-0.86; aPR 0.66; 95% CI 0.42–1.02). Participants from urban areas were 68% more likely to test positive with anti-HEV IgG if they lived in a house rather than an apartment (aPR 1.68; 95% CI 1.22–2.31). The significantly lower percentage of HEV-positive persons was observed among hunters who lived in a city and hunted abroad (aPR 0.54; 95% CI 0.3–0.95). A higher HEV seroprevalence was observed among hunters who preferred to hunt wild boar. However, a lower seroprevalence was observed among cat owners (aPR 0.58; 95% CI 0.34–0.99) (Table 3).

**Table 3:** Seroprevalence of anti-HEV IgG and risk factors associated with HEV infection among hunters living in urban area

Variable	No. of subjects	No. of positive (%)	95% CI	PR (95% CI)	*p*	APR (95% CI)	*p*
Age (years) (*n* = 598)							
≤34	74	18 (24.3)	14.93–27.47	Reference		Reference	
35–49	161	26 (16.1)	15.33–24.74	0.67 (0.39–1.14)	0.133	0.52 (0.31–0.86)	**0.011**
50–64	272	54 (19.9)	18.59–26.58	0.82 (0.52–1.31)	0.394	0.66 (0.42–1.02)	**0.058**
≥65	91	28 (30.8)	22.23–40.1	1.27 (0.77–2.1)	0.363	0.93 (0.58–1.51)	0.754
Type of accommodation							
Flat	279	45 (16.1)	12.30–20.73	Reference		Reference	
House	319	81 (25.4)	21.80–28.30	1.58 (1.14–2.19)	**0.007**	1.68 (1.22–2.31)	**0.002**
Hunting abroad (*n* = 597)							
No	510	113 (22.2)	20.10–25.71	Reference		Reference	
Yes	87	12 (13.8)	11.01–24.67	0.63 (0.36–1.08)	**0.091**	0.54 (0.3–0.95)	**0.031**
Average annual wild boars hunting (on a five-point scale) (*n* = 576)							
1	61	8 (13.1)	10.12–25.58	Reference		Reference	
2	75	18 (24)	20.90–37.03	1.83 (0.86–3.92)	0.12	1.92 (0.9–4.11)	0.093
3	105	21 (20)	15.71–28.57	1.53 (0.72–3.24)	0.271	1.75 (0.84–3.68)	0.14
4	122	34 (27.9)	19.75–31.89	2.13 (1.05–4.31)	**0.036**	2.4 (1.19–4.85)	**0.015**
5	213	42 (19.7)	17.28–25.88	1.51 (0.75–3.03)	0.254	1.7 (0.85–3.39)	0.139
Having a cat (*n* = 585)							
No	490	111 (22.7)	20.27–26.40	Reference		Reference	
Yes	95	13 (13.7)	15.00–25.42	0.61 (0.36–1.03)	0.063	0.58 (0.34–0.99)	**0.042**

Significant* P* values (< 0.05) are in bold

PR (prevalence ratio), aPR (adjusted aPR for age, place of accommodation, hunting abroad, average annual wild boars hunting, having a cat)

The seroprevalence among hunters living in rural areas increased with age; however, this was not statistically significant (*p* = 0.098). The multivariable analysis indicated that people living in rural areas who reported direct contact with farm animals had anti-HEV IgG 66% more frequently than those hunters who did not have similar contact (aPR 1.66; 95% CI 1.12–2.46). Moreover, the seroprevalence of anti-HEV IgG among people who prepared and consumed stewed offal was significantly higher (aPR 1.81; 95% CI 1.29–2.53). Results of univariable analysis indicated that being a farmer may have a significant impact on the positive HEV–IgG test result. However, multivariable analysis did not confirm the above results (PR 1.68; 95% CI 1.12–2.52 vs. aPR 1.24; 95% CI 0.77–1.98) (Table 4).

**Table 4:** Seroprevalence of anti-HEV IgG and risk factors associated with HEV infection among hunters living in rural area

Variable	No. of subjects	No. of positive (%)	95% CI	PR (95% CI)	*p*	APR (95% CI)	*p*
Age (years) (*n* = 423)							
≤34	100	18 (18)	11.03–26.95	Reference		Reference	
35–49	133	32 (24.1)	17.07–32.23	0.27 (0.8–2.24)	0.27	1.34 (0.83–2.18)	0.24
50- 64	170	45 (26.5)	20.01–33.77	0.121 (0.91–2.4)	0.121	1.32 (0.83–2.1)	0.25
> = 65	20	6 (30)	11.89–54.28	0.205 (0.76–3.68)	0.205	1.36 (0.64–2.89)	0.428
Professional contact with animals (*n* = 423)							
No	301	56 (18.6)	14.37–23.50	Reference		Reference	
Yes	122	45 (36.9)	28.33–46.09	0 (1.43–2.77)	**0.000**	1.66 (1.12–2.46)	**0.013**
Cultivating the cereals (*n* = 420)							
No	259	56 (21.6)	17.98–23.98	Reference		Reference	
Yes	161	45 (28)	21.07–31.69	0.138 (0.93–1.82)	0.138	1.05 (0.73–1.51)	0.819
Prepared and consumed stewed offal (*n* = 407)							
No	336	71 (21.1)	16.90–25.89	Reference		Reference	
Yes	71	28 (39.4)	28.03–51.75	0.001 (1.31–2.67)	**0.001**	1.81 (1.29–2.53)	**0.001**
Farmer (*n* = 421)							
No	370	82 (22.2)	18.03–26.74	Reference		Reference	
Yes	51	19 (37.3)	24.12–51.92	0.012 (1.12–2.52)	**0.012**	1.24 (0.77–1.98)	0.386

Significant* P* values (< 0.05) are in bold

PR (prevalence ratio), aPR (adjusted aPR for age, professional contact with animals, cultivating the cereals, prepared, and consumed stewed offal farmer)

## Discussion

This is the first large-scale study considering HEV seroprevalence in Poland. Hunters are a fairly large group which represents a cross section of Polish society. However, this particular group is also occupationally exposed to HEV reservoirs and consists mainly of men, thus preventing the extrapolation of the obtained results to the general population. The advantage of this study is that the examined population constitutes nearly 1% of the source population.

Anti-HEV IgG was identified in 227 Polish hunters (22.2% of the examined group). However, in the publication by Sadkowska-Todys et al. which concerned the same study group, seroprevalence was assessed at 20.3% [25]. Discrepancies between those results are associated with different case study definitions used in the analysis. Nevertheless, the difference between seroprevalence was minor. Other studies performed among the general population and blood donors found higher anti-HEV seroprevalence in a group of hunters compared to non-hunters [26–28]. Authors of those studies hypothesized that hunting activities can be associated with HEV infection. Hunting can result in direct contact with wild boars, considered to be important reservoirs of HEV infections in humans. HEV infection can occur during evisceration of an infected animal, through contact with its blood or faeces. Butchers and slaughterhouse workers were proven to have higher seroprevalence compared with the general population, what can confirm that cutting up carcasses might be a risk factor of HEV infection [29]. These hypotheses are supported by the appearance of HEV RNA in wild boars’ sera and faeces in many European countries: Belgium [30], Estonia [31], Germany [32, 33], Netherlands [34], Slovenia [35], Sweden [36], Italy [37], and Portugal [38]. Moreover, there was also a chronic HEV-3 infection in wild boars described in Germany. It was observed that viremia and fecal excretion lasted more than 5 months [39]. The percentage of seroprevalence in wild boars in Europe amounts from 10.2% in Italy [40] to 47.2% in Spain [15]. Hunting traditions and habits can also lead to an infection [38]. According to the Hunting Act of October 13, 1995, after shooting their first animal, new hunters involve in a traditional ceremony called the “baptism of fire”, during which the hunter's forehead is being covered with blood.

Other conducted studies did not identify hunting activity as an important risk factor. Our result of seroprevalence of anti-HEV IgG is similar to the one obtained among German hunters (21%) after applying the same test. This study revealed no significant difference of HEV seroprevalence between the examined group of hunters and the general population (21 vs. 17%) [41]. Unfortunately, such comparison cannot be made in the presented study due to lack of research among general population in Poland. Other studies hypothesize that professional contact with swine is a stronger risk factor than hunting [31, 42]. Similarly, our study also proved that direct contact with farm animals is an important factor influencing the prevalence of anti-HEV IgG. Hunters that had professional contact with farm animals tested positive with anti-HEV IgG more often than the reference group by 66%. This result is consistent with the knowledge that domestic pigs are proven HEV reservoirs in industrialized countries [13]. Teixeira et al. found significantly higher anti-HEV IgG seroprevalence in workers occupationally exposed to swine compared to general population [29]. It was also established that the frequency of anti-HEV appearance is higher among veterinarians and pig farmers [43–47]. However, this was not observed among Finnish veterinarians specializing in swine diseases [48].

Although regional differences in HEV seroprevalence between Polish provinces were observed in this study, they are difficult to interpret. In Poland, regional discrepancy in seroprevalence was determined in the wild boar population [21]. However, those differences were not reflected in the seroprevalence among hunters. Furthermore, they can be caused by a number of factors, e.g., eating habits or age distribution of the examined population. Nevertheless, those findings should be analyzed with caution due to the small number of participants and the convenience sampling applied in this study.

In our study, the highest frequency of the antibodies occurrence was observed in the oldest age group (over 65 years) (30.6%, *p* value = 0.013). The prevalence among countryside residents increased with age, but this correlation was not statistically significant (*p* = 0,098). Those results were in line with other seroprevalence reviews conducted within the general population, particularly in blood donors’ groups [49–51]. Higher seroprevalence among older people can be a consequence of longer potential exposure. However, in some studies, the relation between age and frequency of HEV infection was not established [27]. We observed that among hunters living in cities, higher seroprevalence was in the youngest (24.3%) and the oldest (30.8%) age groups. Increased seroprevalence in the younger group could be an indirect evidence of hunting as a risk factor. City residents rarely have professional contact with domestic pigs, thus hunting could have the strongest influence on the seroprevalence in this group. It could also be hypothesized that higher seroprevalence can result from increased hunting frequency among younger hunters.

This study revealed higher seroprevalence of HEV among men compared to women (22.5 and 8.3% respectively). However, this difference was not statistically significant due to a limited number of women participants. Higher seroprevalence observed among men in comparison with women was also found in other studies among blood donors and other groups [51–53].

Significant differences were observed depending on the type of accommodation. Participants living in houses were more likely to have anti-HEV antibodies than those who lived in an apartment. Hypothetically, this could be related to owning private springs or wells when living in a house. In a study conducted in France, possible risk factors among the indigenous cases included water consumption from a personal water supply [54].

Zoonotic transmission of HEV is proven to be directly related to consumption of meat, offal, and other products from infected animals. HEV transmission through consumption of wild boar and deer meat has been reported in Japan [55–57], Spain [58], and Italy [59]. Case–control study conducted in Germany found that offal and wild boar meat consumption was a risk factor for hepatitis E infection [60]. Furthermore, consumption of game meat and offal has been associated with higher seroprevalence of HEV infection in humans in other studies [26, 51]. One of the determinants identified in the present study as being related to higher HEV seroprevalence was preparing and consuming stewed offal of hunted animals (aPR 1.6; 95% CI 1.27–2.03). Thermal processing of meat/offal plays a significant role in viral inactivation. To completely inactivate HEV, pork products need to be heated to an internal temperature of 71 °C for 20 min [61]. The present study did not establish any relation between consumption of raw or grilled meat/offal and higher HEV seroprevalence. This could be explained by the fact that there is no tradition of eating wild boar meat/offal without proper heat treatment in Poland (although sausages and ham smoked in low temperatures are popular in some regions). This study did not reveal any connection between consumption of smoked products, regardless of the temperature of the process (cold/hot smoking). However, minced raw meat from roe, deer, and fallow deer is often consumed. Since no specific antibodies against HEV were found in the studies focusing on *Cervidae* population in Poland, it might not be an important factor for HEV sources [21].

The presented study also revealed determinants associated with lower seroprevalence in the studied group of hunters.

Owning a cat (aPR 0.58, 95% CI 0.34–0.99) was found to be related with lower seroprevalence of anti-HEV among hunters living in cities. In the study by Lewis et al., conducted in England and Wales, 60% of cases of hepatitis E declared having pets [62]. An indirect evidence that cats could be a reservoir of HEV is a case of HEV infection in an owner of an anti-HEV-positive cat [63]. Moreover, specific anti-HEV antibodies were found in cats during other studies [64]. However, a research conducted among Austrian soldiers and civilians contradicts those findings. In this study, cat owners presented lower frequency of anti-HEV than the reference group by 61% (OR = 0.61, 95% CI 0.40–0.93) [65]. This is consistent with results of the present study. Another study conducted among pet veterinarians has shown that there are no significant differences in HEV seroprevalence between these professionals and general population (9.9 vs. 13.3%) [66]. Owning a cat was also found to be a protective factor (OR = 0.2; 95% CI 0.06–0.73) in case–control study conducted in Germany [60].

The presented study established a significant relation between washing hands after disembowelling carcasses and lower frequency of HEV seroprevalence (aPR 0.53; 95% CI 0.37–0.74). These findings are consistent with previous observations by Schielke et al. HEV seroprevalence proved to be lower among hunters using gloves during disembowelment than among those who used gloves rarely or never (aPR 0.12; 95% CI 0.02–0.8) [40]. Similarly, Chaussade et al. found that wearing boots at work by pig farm workers and forestry workers were associated with significantly lower seroprevalence of HEV (OR 0.45, *p* = 0.004) [67]. The protective effect of gloves and proper hand hygiene during evisceration indirectly proves that direct contact with animal reservoirs could be a risk factor of HEV infection [40, 41]. The lack of personal protection could mean a low awareness of exposure to potential infectious agents among those hunters. Implementing those simple protection measures can minimize the risk of an infection.

The appearance of only anti-HEV IgM indicates an active infection [68]. The hunters that tested positive for anti-HEV IgM did not report any symptoms indicating acute hepatitis. In addition, three of them were positive with both anti-HEV IgM and IgG. Although that HEV RNA has not been detected, an active asymptomatic infection cannot be excluded [69]. However, a study among German blood donors indicates that diagnostic window between identifying HEV RNA and the first detection of specific anti-HEV antibodies lasts from 8 to 48 days (depending on the test used) [70].

The results of this study should be interpreted in the context of its limitations. One of the main limitations is the classification of participants as cases based on the results from the EIA test–anti-HEV IgG, what can result in misclassification. Moreover, the study was conducted applying version of recomWell IgM and IgG test and recomLine IgM/IgG (Mikrogen, Germany) available in Poland in 2011. The tests were later improved to achieve higher sensitivity [71, 72]. The present study bears the risk of underestimation of viremia in the examined group of hunters. Especially, only 5 of the 1021 studied hunters were tested for HEV RNA. Authors are aware that convenience sampling of participants provided lower representativeness of the study sample in the entire population of hunters in Poland. However, acquiring a large study group allowed to minimize the possible error. Another limitation of the study was volunteer bias. Although knowledge about HEV infection among hunters is small, this study also assessed the seroprevalence of other foodborne diseases. Hunters are usually familiar with risk factors associated with trichinellosis and echinococcosis and, therefore, could be more interested in participating in the study. Construction of the question included in our survey, which referred only to the general “wild game meat”, did not enable to establish what species it actually indicated.

Due to high seroprevalence of HEV among hunters, a large-scale study in the general population and/or selected risk groups is recommended to estimate the actual scale of this problem in Poland. Results of this study do not exclude hunting activity as a factor linked to HEV infection. However, it has significantly smaller importance when compared with other risk factors, such as professional contact with farm animals and consumption of offal from animal reservoirs. An information campaign should be organised among hunters to spread the knowledge of HEV and promote proper protective measures.
